# On Abstinence from Drinks in the Treatment of Some Diseases

**Published:** 1846-04

**Authors:** 


8. *On Abstinence from Drinks in the Treatment of some dis seases.*—Dr. Bourge thinks that the quantity of drink administered in disease is not a matter of indifference, that it ought to be regulated by the medical attendant, and should not be left to the taste of the patient or the caprice of the nurse.

Thus, in all affections accompanied by a predominance of the serous or aqueous element in the blood, this physician thinks that we ought to diminish very much the usual quantity of drink, or even to suppress it altogether. Such are, for example, cases of dropsy, profuse sweatings, chlorosis, suffocative catarrh, abundant diarrhœas, diabetes, organic diseases of the heart, and, finally, asthma, whenever this malady owes its existence to a pathological condition of the central organ of the circulation.—*Dublin Hospital Gazette*, in *Boston Medical and Surgical Journal.*

